# Childhood maltreatment and non-suicidal self-injury in college students: **chain** mediation by alexithymia and depression along with a symptom-level network analysis

**DOI:** 10.3389/fpsyg.2026.1751101

**Published:** 2026-07-08

**Authors:** Jiemei Wang, Feng Gong, Qiaodan Gan, Huazhen He, Xinyu Li, Tianxiang Xu, Xinran Yang, Yunyi Hu

**Affiliations:** 1 Mental Health Education and Counseling Center, Nanning University, Nanning, China; 2 School of Information Resource Management, Renmin University of China, Beijing, China; 3 School of Marxism, Central South University, Changsha, China; 4 School of Computer Science and Engineering, Central South University, Changsha, China; 5 School of Software and Microelectronics, Peking University, Beijing, China

**Keywords:** alexithymia, childhood maltreatment, college students, depression, network analysis, non-suicidal self-injury

## Abstract

**Background:**

Childhood maltreatment is a well-established risk factor for various mental health and behavioral problems, including non-suicidal self-injury (NSSI). Despite its prevalence, the associations between childhood maltreatment and NSSI, particularly as mediated by alexithymia and depression, remain not fully explored. This study primarily employs structural equation modeling (SEM) to test the hypothesized relationships between childhood maltreatment, alexithymia, depression, and NSSI, and additionally incorporates network analysis to examine the symptom-level connections among these constructs at the micro level, complementing the macro-level insights from SEM.

**Method:**

We recruit a sample of 1,414 college students. Participants complete the Childhood Trauma Questionnaire-Short Form (CTQ-SF), the Toronto Alexithymia Scale (TAS-20), the Self-Rating Depression Scale (SDS), and the NSSI Behavior Assessment Questionnaire. We then employ SEM to test the hypothesized mediation model and use network analysis to visualize the interconnections among the specific dimensions of childhood maltreatment, alexithymia, depression, and NSSI.

**Results:**

SEM results indicate that alexithymia and depression significantly mediate the relationship between childhood maltreatment and NSSI, forming a chain mediating effect. Network analysis identifies “Difficulty identifying feelings” (DIF) as the most central node in the network. Moreover, DIF, NSSI, and Affective Symptoms (AS) were identified as key bridge symptoms connecting the clusters within the network.

**Conclusion:**

Our findings highlight the importance of focusing on the emotional processing deficit and emotional problems in the intervention and prevention of NSSI among college students.

## 1 Introduction

Non-suicidal self-injury (NSSI) is defined as the direct and deliberate destruction of one's own body tissue without suicidal intent (Zetterqvist et al., 2013). The diagnostic and statistical manual of mental disorders (5th ed.; DSM-5) lists NSSI as a distinct diagnostic category, highlighting its status as a major global public health issue. Approximately 30% to 35% of college students first engage in NSSI after the age of 18 (Gu et al., 2019). This indicates that this period is an important stage for personal growth and exploration, and also a period when NSSI vulnerability increases (Barnett et al., 2024; Zou et al., 2024). For individual development, NSSI constitutes a significant maladaptive behavior, frequently co-occurring with severe psychological distress and closely associated with adverse physical and mental health outcomes (Kiekens et al., 2018, 2023). For society, NSSI has become a widespread public health challenge demanding effective intervention and prevention strategies (Buerger et al., 2022; WHO., 2020). Therefore, a deeper investigation into the factors related to NSSI among college students is crucial for protecting their wellbeing and preventing these harmful consequences.

### 1.1 The relationship between childhood maltreatment and NSSI

Childhood maltreatment (CM), as a core component of adverse early experiences, typically refers to acts or omissions perpetrated by parents or other caregivers before the age of 18 that significantly increase an individual's risk of physical and mental health impairment (Gilbert et al., 2009; Mattar et al., 2025). Childhood maltreatment primarily encompasses five forms: emotional neglect (EN, failure to meet a child's emotional and psychological needs), physical neglect (PN, failure to provide essential material and safety conditions), emotional abuse (EA, involving belittling, threatening, or humiliating behavior), physical abuse (PA, acts causing bodily harm or pain), and sexual abuse (SA, involving sexual acts or exploitation of a child) (Johnson and McKernan, 2021). Extensive empirical research has identified childhood maltreatment as a significant risk factor for NSSI. For instance, studies from multiple countries, including Germany, Canada, Australia, the United States, and China, consistently show a strong link between childhood maltreatment and later engagement in NSSI (Li W. et al., 2024; Paul and Ortin, 2019; Wu et al., 2023). According to Yates's (2004) developmental psychopathology framework, NSSI can function as a maladaptive coping strategy to compensate for five adaptive capacities impaired by early trauma: motivational, attitudinal, instrumental, emotional, and interpersonal skills. Nock et al. (2010) experiential avoidance model posits childhood maltreatment as a distal risk factor for NSSI that heightens vulnerability by impairing healthy emotional, cognitive, and interpersonal development. College students occupy a crucial transitional phase between adolescence and adulthood, where early maltreatment experiences may disrupt self-identity formation, emotional regulation, and interpersonal relationship development, giving rise to problematic behavior (Hailes et al., 2019; Park, 2025). Furthermore, previous research has indicated that childhood maltreatment elevates the risk of NSSI by 2.7 to 6.1 times, with all maltreatment types showing significant associations with NSSI among college students (Iskric et al., 2020). Based on this, we propose the following hypothesis:

Hypothesis 1: Childhood maltreatment may be positively associated with NSSI in college students.

In addition, different theoretical models explain how childhood maltreatment leads to NSSI by focusing on different aspects of the problem. The cumulative risk model suggests that various forms of maltreatment often co-occur and that their combined, additive effect increases the risk of NSSI (Afifi et al., 2016). For example, a longitudinal study applying this model found that higher cumulative abuse scores were linked to a greater incidence of NSSI, indicating a cumulative risk effect (Rong et al., 2024). In contrast, the multiple independent risk model proposes that while types of maltreatment frequently co-occur, each specific form can also contribute independently to negative outcomes. For instance, sexual and physical abuse may increase the risk of NSSI by reducing pain sensitivity. This is supported by findings that individuals who engage in NSSI may have higher pain thresholds (Cummins et al., 2025; Schönfelder et al., 2021). Similarly, emotional abuse and neglect are pervasive yet often overlooked forms of maltreatment. They pose a lasting threat to mental health and are strongly associated with both suicide and NSSI (Cao et al., 2024; Schönfelder et al., 2019), a link confirmed by longitudinal research showing they predict NSSI in adulthood (Zhang R. et al., 2024). Therefore, an integrated perspective that combines macro-level and micro-level analyses, while also distinguishing between different forms of abuse, is essential for developing effective NSSI prevention and intervention strategies.

### 1.2 The associations among childhood maltreatment, alexithymia, and NSSI

Alexithymia refers to difficulty in recognizing and expressing emotions, encompassing three core dimensions: difficulty identifying feelings (DIF), difficulty describing feelings (DDF), and externally oriented thinking (EOT) (Preece et al., 2024). Childhood maltreatment is widely recognized as a risk factor for alexithymia. Early-life abuse has been linked to disruptions in affective schemata development (Ardizzi et al., 2025) and to higher alexithymia levels in adulthood (Wang, 2024). Children from abusive homes often experience emotional invalidation, a context associated with learned helplessness, and limited exposure to healthy emotional expression co-occurs with poor emotional awareness and difficulty expressing feelings (Ditzer et al., 2023; Luminet et al., 2021). Compared to non-abused individuals, those with maltreatment histories report lower self-esteem (Zhang et al., 2023) and endorse fewer adaptive emotion regulation strategies (Clinchard et al., 2025; O'Mahen et al., 2015), patterns that may contribute to the emergence of alexithymia (Preece et al., 2023) and subsequent emotional regulation difficulties (Preece et al., 2017).

Experiential avoidance theory posits that NSSI may function as a coping mechanism for unpleasant stimuli (Nock et al., 2010). Individuals with high alexithymia are more likely to use NSSI to avoid such stimuli or as a substitute for emotional expression (Hasking et al., 2017). Extensive research confirms a strong association between affective disorders and NSSI (Iskric et al., 2020), evident across diverse populations. In clinical adolescent samples, alexithymia is common and serves as a pathway through which psychological distress is expressed via NSSI (Dong et al., 2023; Raffagnato et al., 2020); it is also highly prevalent among adolescent outpatients with NSSI (Gatta et al., 2016). In the general population, individuals with NSSI histories exhibit higher rates of affective disorders (Greene et al., 2021), and longitudinal data indicate that adolescent affective disorders are associated with both immediate and later NSSI (Garisch and Wilson, 2015). Although NSSI contradicts the self-preservation instinct, it may provide temporary relief from negative emotions (Danese and Widom, 2023). Therefore, we propose the following hypothesis:

Hypothesis 2: Alexithymia may mediate the relationship between childhood maltreatment and NSSI among college students.

### 1.3 The associations among childhood maltreatment, depression, and NSSI

Depression is a common mental disorder that severely impairs psychosocial functioning (Malhi and Mann, 2018). Substantial evidence indicates that childhood abuse experiences are strongly associated with depression. For example, a meta-analysis demonstrated a significant association between emotional abuse, neglect, and depressive disorders (Norman et al., 2012). Longitudinal research has shown that early-life maltreatment is associated with depression several years later (Jessar et al., 2017), and individuals with a history of childhood abuse report significantly higher levels of depression than those without (Tang et al., 2024). As a distal risk factor, childhood maltreatment is theorized to increase susceptibility to depression through its association with impaired emotional regulation (Humphreys et al., 2020).

Depression, in turn, is strongly associated with NSSI. Research indicates that depression co-occur with an elevated risk of NSSI. Shen et al. (2023) identified a strong correlation between adolescent depression and NSSI in clinical samples, noting that emotion regulation served as the primary function of NSSI for these individuals. Depression involves persistent distress from intense negative emotions, such as low mood, numbness, and suffering (Huang et al., 2024; Pomerantz and Rudolph, 2003). According to the experiential avoidance model, individuals are strongly motivated to escape intolerable emotional states (Nock et al., 2010). Consistent with this framework, for individuals with depression, NSSI may function as a form of negative reinforcement, providing temporary relief from emotional distress (Shen et al., 2023). Extending this line of research, studies have examined the mediating role of depression in the association between childhood maltreatment and NSSI. For example, one study of female adolescents found that depression fully mediated the link between childhood maltreatment and NSSI (Shenk et al., 2010). A longitudinal study by Zhang R. et al. (2024) revealed that depression played a mediating role in the association between emotional abuse and NSSI. Therefore, we propose the following hypothesis:

Hypothesis 3: Depression may mediate the association between childhood maltreatment and NSSI among college students.

### 1.4 The associations among alexithymia, depression and NSSI

Alexithymia and depression demonstrate a well-established comorbidity (Xie et al., 2024), with specific alexithymia dimensions showing positive correlations with the severity of depressive symptoms (Kieraité et al., 2024; Preece et al., 2024). Some researchers even argue that measures of alexithymia and depression are structurally indistinguishable (Marchesi et al., 2014). We propose that alexithymia may serve as a precursor to depression. The cognitive developmental theory of emotional awareness posits that emotional awareness is essentially the ability of individuals to conceptualize and express their own emotions as well as those of others. It follows a progressive five-level cognitive developmental hierarchy, and deficits in this ability can directly lead to difficulties in emotional adaptation and psychological regulation (Lane and Schwartz, 1987; Xie et al., 2024). Meanwhile, extensive psychological research has confirmed that the occurrence and development of the majority of psychological disorders, including depression, are rooted in impairments in emotional processing and cognitive regulation mechanisms (Li Y. et al., 2025; Lu et al., 2026). Within this theoretical framework, alexithymia, conceptualized as a comprehensive deficit in emotional awareness, may increase individuals' vulnerability to depression, making them more prone to persistent emotional distress and maladaptive coping styles when facing stress, conflicts, or adverse life events (Li T. et al., 2025; Morie et al., 2024). Individuals with higher levels of alexithymia tend to be more passive and negative when confronting stressful events, and are more likely to adopt maladaptive strategies such as suppression, self-blame, and catastrophizing, thereby further exacerbating the occurrence and development of depression (Kieraité et al., 2024; Valentine et al., 2024). A study by Lu et al. (2024) found that alexithymia serves as a coping mechanism for individuals with a history of childhood physical abuse, protecting themselves from further psychological harm by numbing their emotions or becoming emotionally distant. However, this emotional suppression impairs the development of emotional awareness and may exacerbate depressive symptoms over time.

The association between depression and NSSI is also well-documented. Research indicates that up to 55.1% of individuals with NSSI are diagnosed with depression (Wang et al., 2021). Similarly, among adolescents with major depressive disorder, approximately 34.2% have a history of NSSI (Kang et al., 2021). These statistics highlight the significant comorbidity between the two conditions. Most studies find a high correlation and comorbidity between depression and NSSI (Zhang et al., 2022). Clinical research indicates that adolescents with depressive disorders are highly likely to employ NSSI to regulate negative emotions and reduce tension tension (Li Y. et al., 2024), and that a single beneficial experience can foster behavioral dependency patterns (Tang et al., 2022). Meta-analyses confirm that depressive symptoms significantly influence NSSI (Cipriano et al., 2017; Valencia-Agudo et al., 2018), which is often used to counteract feelings of numbness and melancholy (Tang et al., 2022). As previously mentioned, according to the experiential avoidance model, individuals are strongly motivated to escape intolerable emotional states (Nock et al., 2010). Consequently, for people with depression, NSSI acts as a form of negative reinforcement, providing relief from emotional distress. They may use self-harm to escape the profound emotional suffering caused by depression (Shen et al., 2023). Therefore, we propose the following hypothesis:

Hypothesis 4: Alexithymia and depression may play a chain mediating role in the association between childhood maltreatment and NSSI among college students.

### 1.5 The present study

Given that NSSI constitutes a major public health concern both in China and globally, further investigation into its underlying mechanisms is warranted. While previous studies have predominantly examined the impact of factors such as childhood maltreatment, alexithymia, and depression on NSSI within separate theoretical frameworks, the present study adopts an integrated framework. Grounded in Yates's (2004) developmental psychopathology framework, we conceptualize childhood abuse as a distal risk factor for NSSI. Concurrently, drawing on Nock et al. (2010) experiential avoidance theory, alexithymia and depression are positioned as proximal risk factors. This study aims to elucidate how distal risk factors contribute to the development of NSSI by shaping emotional and cognitive processes. Furthermore, integrating the cognitive developmental theory of emotional awareness (Lane and Schwartz, 1987), we investigate the chain-mediated role of alexithymia and depression in the relationship between childhood maltreatment and NSSI.

Although previous studies have examined the mediating roles of alexithymia and depression separately in the relationship between childhood maltreatment and NSSI, typically using total scale scores in correlation or regression analyses. Traditional structural equation modeling (SEM) can test *a priori* path hypotheses at the macro construct level, but this approach may overlook the complex interconnections among specific dimensions of these constructs. Consequently, it remains unclear how different types of childhood maltreatment, specific facets of alexithymia, and depression are interrelated. In contrast, the network theory of psychopathology conceptualizes mental disorders as complex systems of interacting symptoms, emphasizing their direct associations to reveal underlying connections (Borsboom and Cramer, 2013; Hofmann and Curtiss, 2018). Thus, network analysis offers a valuable complement to macro-level SEM findings.

Accordingly, this study has two primary objectives. First, we will use SEM to examine the associations among childhood maltreatment, alexithymia, depression, and NSSI. Second, we will construct a symptom network as an exploratory analysis to analyze the interrelationships among different types of childhood maltreatment, specific dimensions of alexithymia, various depressive symptoms, and NSSI. The aim is to identify central symptoms that link these variables to NSSI, thereby providing exploratory insights for intervention strategies.

## 2 Method

### 2.1 Measures

#### 2.1.1 Childhood trauma questionnaire-short form, CTQ-SF

Childhood maltreatment was assessed using the Chinese version of the CTQ-SF (Bernstein et al., 1998; Zhao et al., 2005), a well-validated 28-item measure that assesses five types of maltreatment experienced before the age of 18: emotional, physical, and sexual abuse (EA, PA, SA), along with emotional and physical neglect (EN, PN). Responses are recorded on a five-point Likert scale, from 1 (never) to 5 (always), where higher scores in each dimension indicate more severe maltreatment. The Chinese version demonstrated good reliability and validity among Chinese college students (Zhang Y. et al., 2024). In this study, the Cronbach's α coefficient for this scale was 0.926.

#### 2.1.2 Toronto alexithymia scale, TAS-20

Alexithymia was assessed using the Chinese version of TAS-20, which was developed by Bagby et al. (1994) and revised by Yi et al. (2003). It comprises three dimensions: difficulty describing one's own feelings (DDF), difficulty identifying one's own feelings (DIF), and externally oriented thinking (EOT). Scoring utilized a 5-point Likert scale ranging from 1 (strongly disagree) to 5 (strongly agree). Higher scores indicate greater levels of alexithymia. The Chinese version demonstrated good reliability and validity among Chinese college students (Liu et al., 2021). In this study, the Cronbach's α coefficient for this scale was 0.903.

#### 2.1.3 Self-rating depression scale, SDS

Depression was assessed using Zung's (1965) SDS. This 20-item instrument was originally designed as a unidimensional measure of depressive severity. However, in validating the scale for Chinese populations, Wang and Tan (2011) identified a four-factor structure comprising: affective symptoms (AS), physiological symptoms (PS), Psychomotor Symptoms (MS), and cognitive symptoms (CS). Scoring employs a 4-point scale ranging from 1 (never or very seldom) to 4 (most or all of the time). Typically, the raw sum of all 20 items is calculated and then multiplied by 1.25 to derive the standardized score. Higher scores indicate more severe depression. Previous research has demonstrated good reliability and validity among Chinese college students (Xu et al., 2025). In this study, the Cronbach's α coefficient for the scale was 0.898.

#### 2.1.4 NSSI behavior assessment questionnaire

NSSI was assessed using the Adolescent NSSI Behavior Assessment Questionnaire (Wan et al., 2018). Although the ANSAQ was initially developed and validated in adolescent populations, its reliability and psychometric stability have been well established in adult populations across numerous studies (Lai et al., 2026; Zheng et al., 2025). This 12-item instrument measures the frequency of NSSI behavior over the preceding year. Participants rate each item on a five-point scale from 0 (never) to 4 (always). Higher total scores indicate a greater frequency of NSSI, with a score of zero signifying no NSSI behavior. In this study, the Cronbach's α coefficient for the scale was 0.947.

### 2.2 Participants and procedure

This cross-sectional study collected data in July 2025 using the Chinese online survey platform, Wenjuanxing. This study has been approved by the ethics committee of the authors' institution and has adhered to relevant ethical guidelines. At the beginning of the questionnaire, participants were presented with the study's objectives, confidentiality principles, and ethics review documentation. Participation was voluntary, and respondents provided informed consent before proceeding. Inclusion criteria included: (1) Aged 17 to 30 years; (2) College students enrolled in Chinese mainland universities, including undergraduate, master's, and doctoral candidates; (3) Provided written informed consent prior to participation. Exclusion criteria included: (1) a completion time of less than 182 s (calculated from a minimum of 2 s per item; (2) failure to pass an attention-check item; (3) providing identical responses across all items. A total of 1,468 questionnaires were distributed, of which 1,414 were valid, resulting in a 96.32% valid response rate.

### 2.3 Statistical analyses

Data analysis was conducted using SPSS 28.0, AMOS 26.0, and R 4.4.1. SPSS was used for preliminary analyses, including descriptive statistics, partial correlation, and analysis of variance. Common method bias was assessed via confirmatory factor analysis (CFA), and SEM was conducted to test the hypothesized mediation model, both using AMOS 26.0. The model specified childhood maltreatment (with its five dimensions: PN, EA, PA, EN, SA) as the independent variable and NSSI as the dependent variable. Alexithymia (with its three dimensions: DDF, DIF, EOT) and depression (with its four dimensions: CS, PS, MS, AS) were included as mediating variables. Model fit was assessed using Hu and Bentler's (1999) criteria: χ^2^/ *df* < 5, CFI > 0.90, TLI > 0.90, and RMSEA < 0.08. To test the mediation effects, a bootstrap analysis with 5,000 samples was conducted to estimate the direct, indirect, and total effects.

Network analysis was performed using R 4.4.1. A network was constructed in which nodes represent symptoms and edges represent regularized partial correlations between them; thicker edges indicate stronger relationships. The EBICglasso estimation method was used with a hyperparameter gamma of 0.5. Densely connected groups of nodes form communities. Bridge edges, which connect these communities, are colored to denote positive (purple) or negative (red) correlations. Node centrality was assessed using expected influence (EI), a metric suitable for networks containing both positive and negative connections. Bridge centrality was also calculated using EI. The stability of the network was evaluated using a case-dropping bootstrap procedure, and the correlation stability coefficient (CS-coefficient) was calculated. Following Epskamp and Fried (2018), a CS-coefficient of at least 0.25 was deemed acceptable, with a value above 0.50 preferred.

## 3 Result

### 3.1 Sample characteristics

The final sample consisted of 1,414 participants after applying exclusion criteria to an initial pool of 1,468 respondents. The sample's demographic characteristics are summarized in Table 1. Participants had a mean age of 21.95 years (SD = 2.45) and included 386 males (27.3%) and 1,028 females (72.7%). In terms of family background, 541 (38.3%) were only children and 873 (61.7%) were not; 720 (50.9%) were from urban areas and 694 (49.1%) from rural areas.

**Table 1:** Demographic characteristics of the participants.

Variables	*n* (%)	NSSI	*t/F*
Sex			
Male	386	5.78 ± 9.05	2.67^**^
Female	1,028	4.42 ± 6.92	
Grade			
Freshman	96	3.93 ± 6.80	24.46^***^
Sophomore	232	9.44 ± 11.44	
Junior	434	4.41 ± 7.40	
Senior	373	4.06 ± 5.61	
Master	261	2.84 ± 4.21	
Doctor	18	1.94 ± 1.55	
Only child			
Yes	541	4.16 ± 6.46	2.61^**^
No	873	5.18 ± 8.18	
Region			
Urban	720	5.29 ± 7.88	2.53^*^
Rural	694	4.27 ± 7.23	
Left-behind experience			
Yes	524	5.51 ± 7.90	2.75^**^
No	890	4.37 ± 7.37	

*N* = 1,414.

^*^p < .05.

^**^p < .01.

^***^p < .001.

### 3.2 Common method bias test

Given the use of self-report data, common method bias was a potential concern (Podsakoff et al., 2024). To address this issue, we first compared the fit of alternative confirmatory factor analysis (CFA) models to assess the discriminant validity of the constructs. Then, we employed the unmeasured latent method construct (ULMC) and Harman's single-factor test to examine common method biases. The alternative confirmatory factor analysis models showed inadequate fit, while the four-factor model demonstrated satisfactory fit, thereby verifying the discriminant validity of the study's core constructs. Subsequently, we introduced a common method variance (CMV) factor into the four-factor baseline model (Table 2). Results indicated that this addition led to only negligible improvements in model fit: RMSEA and SRMR decreased by 0.002 and 0.005, respectively, while TLI and CFI increased by merely 0.004 and 0.002 (all changes < 0.01). Furthermore, Harman's single-factor test revealed 12 factors with eigenvalues exceeding 1.0, with the first factor accounting for only 27.32% of the variance, below the recommended 50% threshold. Thus, common method bias did not pose a substantial threat in this study.

**Table 2:** Confirmatory factor analysis.

Model	χ^2^	*df*	χ^2^/*df*	RMSEA	SRMR	TLI	CFI
Single-factor model	34,083.666	2,853	11.947	0.168	0.139	0.475	0.488
Two-factor model	24,330.422	2,851	8.534	0.131	0.088	0.639	0.648
Three-factor model	19,856.673	2,849	6.970	0.086	0.074	0.842	0.879
Four-factor model	17,666.519	2,846	6.207	0.061	0.060	0.919	0.933
Four-factor model + CMV	16,674.049	2,773	6.013	0.059	0.055	0.923	0.935

In the one-factor model, all four variables were loaded onto a single latent factor. In the two-factor model, childhood maltreatment and NSSI were combined into one latent factor, while alexithymia and depression were combined into another. In the three-factor model, alexithymia and depression were combined into one latent factor, with childhood maltreatment and NSSI each specified as separate factors. In the four-factor model, all four variables were specified as distinct latent factors. CMV incorporated a common method variance (CMV) latent factor.

### 3.3 Correlation analysis

After controlling for demographic variables (sex, age, grade, region, left-behind experience, and only-child status), partial correlation analyses were conducted to examine the relationships between childhood maltreatment, alexithymia, depression, and NSSI (see Table 3). The results indicated that childhood maltreatment was positively correlated with alexithymia (*r* = 0.34, *p* < 0.001), depression (*r* = 0.32, *p* < 0.001), and NSSI (*r* = 0.58, *p* < 0.001). Thus, hypothesis 1 was supported. Alexithymia was also significantly correlated with depression (*r* = 0.71, *p* < 0.001) and NSSI (*r* = 0.50, *p* < 0.001). Similarly, depression showed a significant positive correlation with NSSI (*r* = 0.49, *p* < 0.001).

**Table 3:** Correlation analysis.

	1	2	3	4	5	6	7	8	9	10	11	12	13	14	15	16
1.Physical neglect (PN)	1															
2.Physical abuse (PA)	0.48^***^	1														
3.Emotional neglect (EN)	0.62^***^	0.49^***^	1													
4.Emotional abuse (EA)	0.57^***^	0.48^***^	0.72^***^	1												
5.Sexual abuse (SA)	0.38^***^	0.61^***^	0.35^***^	0.34^***^	1											
6.Childhood maltreatment	0.78^***^	0.76^***^	0.84^***^	0.83^***^	0.66^***^	1										
7.Difficulty describing one's own feelings (DDF)	0.21^***^	0.17^***^	0.27^***^	0.25^***^	0.19^***^	0.28^***^	1									
8.Difficulty identifying one's own feelings (DIF)	0.28^***^	0.27^***^	0.26^***^	0.31^***^	0.25^***^	0.35^***^	0.80^***^	1								
9.Externally oriented thinking style (EOT)	0.23^***^	0.16^***^	0.20^***^	0.18^***^	0.19^***^	0.25^***^	0.59^***^	0.60^***^	1							
10.Alexithymia	0.28^***^	0.24^***^	0.27^***^	0.29^***^	0.24^***^	0.34^***^	0.89^***^	0.80^***^	0.78^***^	1						
11.Affective symptoms (AS)	0.33^***^	0.22^***^	0.34^***^	0.37^***^	0.21^***^	0.36^***^	0.54^***^	0.57^***^	0.40^***^	0.57^***^	1					
12.Psychomotor symptoms (MS)	0.20^***^	0.14^***^	0.23^***^	0.24^***^	0.15^***^	0.25^***^	0.58^***^	0.56^***^	0.51^***^	0.62^***^	0.63^***^	1				
13.Physiological symptoms (PS)	0.26^***^	0.17^***^	0.27^***^	0.28^***^	0.20^***^	0.31^***^	0.60^***^	0.59^***^	0.52^***^	0.65^***^	0.70^***^	0.69^***^	1			
14.Cognitive symptoms (CS)	0.19^***^	0.12^***^	0.24^***^	0.23^***^	0.14^***^	0.22^***^	0.56^***^	0.51^***^	0.57^***^	0.62^***^	0.52^***^	0.67^***^	0.66^***^	1		
15.Depression	0.26^***^	0.18^***^	0.28^***^	0.29^***^	0.22^***^	0.32^***^	0.66^***^	0.64^***^	0.59^***^	0.71^***^	0.76^***^	0.82^***^	0.92^***^	0.86^***^	1	
16.NSSI	0.49^***^	0.47^***^	0.41^***^	0.45^***^	0.51^***^	0.58^***^	0.40^***^	0.47^***^	0.45^***^	0.50^***^	0.43^***^	0.38^***^	0.45^***^	0.42^***^	0.49^***^	1
*M*	7.58	7.17	9.36	8.88	6.70	39.71	13.95	18.16	22.67	54.78	4.67	4.94	18.28	20.30	60.22	4.79
SD	3.34	3.05	4.17	3.77	2.83	13.67	4.20	6.39	4.33	13.30	1.52	1.42	4.75	3.86	12.79	7.59

*N* = 1,414.

^***^p < 0.001. *M* = mean. SD = standard deviations.

### 3.4 Structural equation model

Before conducting SEM, we examined the potential for multicollinearity between alexithymia and depression, as these two variables were strongly correlated (*r* = 0.71). Multicollinearity diagnostics indicated that both alexithymia and depression had variance inflation factor (VIF) values of 2.184, below the conventional cutoff of 5.0. Having ruled out significant multicollinearity concerns, we next sought to establish the discriminant validity of these two constructs, given their substantial correlation. To empirically test whether these two constructs were distinguishable in our data, we conducted a confirmatory factor analysis comparing a two-factor model with a one-factor model. The results indicated that the two-factor model (χ^2^/ *df* = 9.41, CFI = 0.97, TLI = 0.95, RMSEA = 0.07) provided a significantly better fit to the data than the one-factor model (χ^2^/ *df* = 12.39, CFI = 0.70, TLI = 0.69, RMSEA = 0.09), supporting the discriminant validity of the two constructs. Finally, given that these factors are significantly correlated with NSSI, they were included as covariates in the analysis, including sex, age, grade, region, left-behind experience, and only-child status.

**Figure 1:**
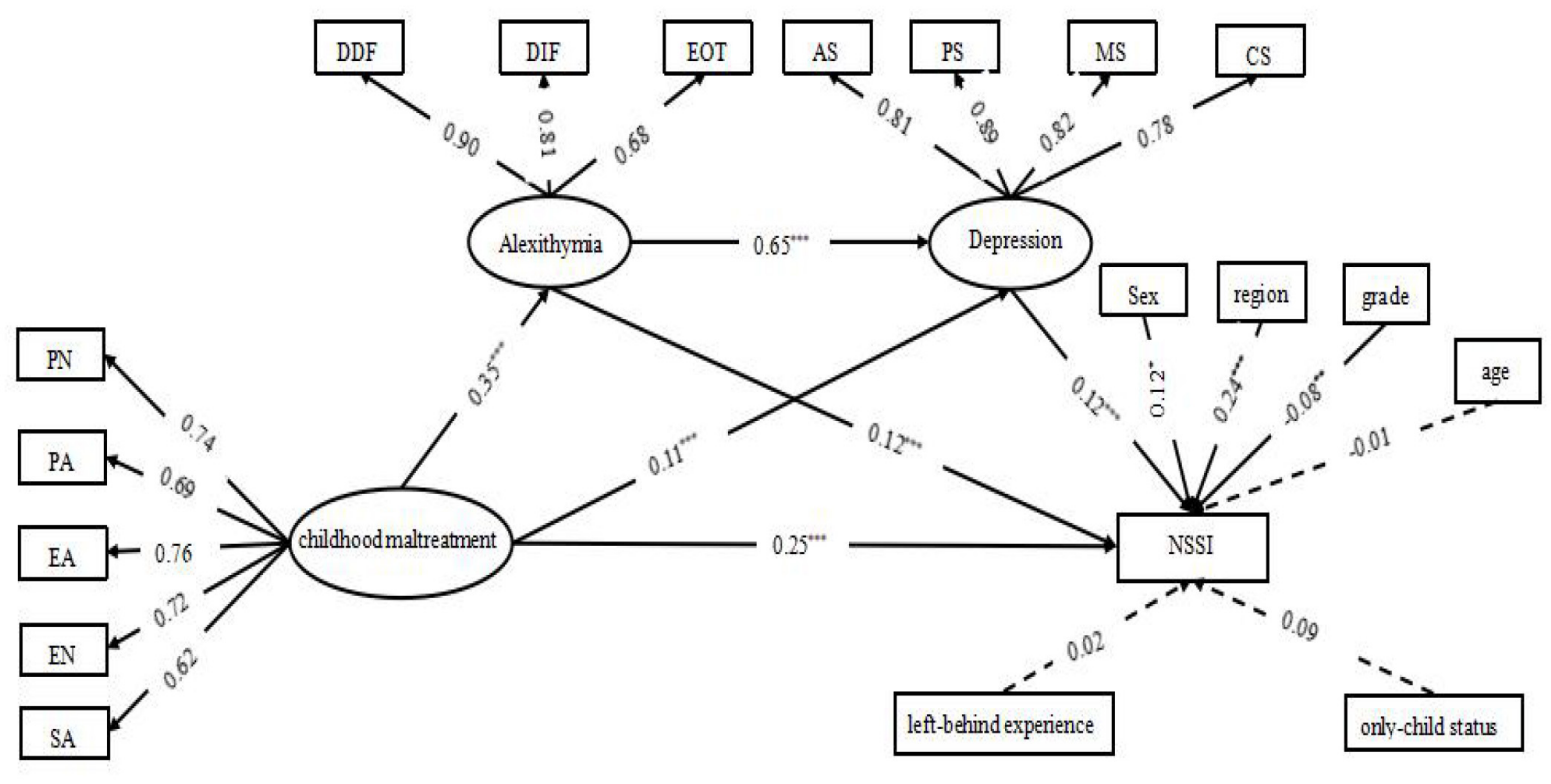
The chain mediation model for childhood maltreatment, alexithymia, depression and NSSI. *p < 0.05. **p < 0.01. ***p < 0.001.

Figure 1 depicts the SEM examining the associations between childhood maltreatment and NSSI among college students, with detailed results provided in Table 4. The model demonstrated satisfactory fit to the data: χ^2^/ *df* = 5.989, CFI = 0.975, TLI = 0.956, RMSEA = 0.069, SRMR = 0.038. Although the χ^2^/ *df* > 5, this remains acceptable for large samples (*N* > 1,000) according to Sathyanarayana and Mohanasundaram (2024). SEM results indicated a significant direct effect of childhood maltreatment on NSSI [β = 0.252, 95% *CI*= (0.230, 0.274)], along with significant indirect effects mediated through alexithymia and depression. Bootstrap analysis with 5,000 samples confirmed significant specific indirect effects through alexithymia [β = 0.042, 95% CI = (0.029, 0.057)], through depression [β = 0.013, 95% CI = [0.008, 0.020]), and through the alexithymia-depression chain [β = 0.027, 95% CI = (0.018, 0.036)]. Therefore, hypotheses 1 to 4 were all supported. The total indirect effect was 0.082 [95% CI = (0.065, 0.100)], and the total effect of childhood maltreatment on NSSI was 0.333 [95% CI = (0.310, 0.356)]. All effects were statistically significant, as indicated by confidence intervals that did not include 0. It should be noted that childhood maltreatment exerts a greater direct effect on NSSI (β = 0.252), while the mediating effects of alexithymia and depression, as well as the chain-mediation effect (β = 0.082), are relatively smaller.

**Table 4:** Childhood maltreatment and NSSI in the mediation effect analysis.

Relationship	Estimate	S.E.	95%CI		*P*	*R^2^*
			Lower	Upper		
Alexithymia						0.189
Childhood maltreatment → alexithymia	0.348	0.024	0.301	0.396	<0.001	
Depression						0.569
Childhood maltreatment → depression	0.109	0.018	0.073	0.145	<0.001	
Alexithymia → depression	0.654	0.019	0.617	0.691	<0.001	
NSSI						0.518
Alexithymia → NSSI	0.121	0.016	0.089	0.152	<0.001	
Depression → NSSI	0.117	0.017	0.085	0.150	<0.001	
Direct effect	0.252	0.012	0.230	0.274	<0.001	
Mediating effect of alexithymia	0.042	0.007	0.029	0.057	<0.001	
The mediating effect of depression	0.013	0.003	0.008	0.020	<0.001	
Chain mediating effect	0.027	0.005	0.018	0.036	<0.001	
Total indirect effect	0.082	0.009	0.065	0.100	<0.001	
Total effect	0.333	0.012	0.310	0.356	<0.001	

*N* = 1,414. Bootstrap sample size = 5,000. *CI* = confidence interval.

### 3.5 Network analysis

**Figure 2:**
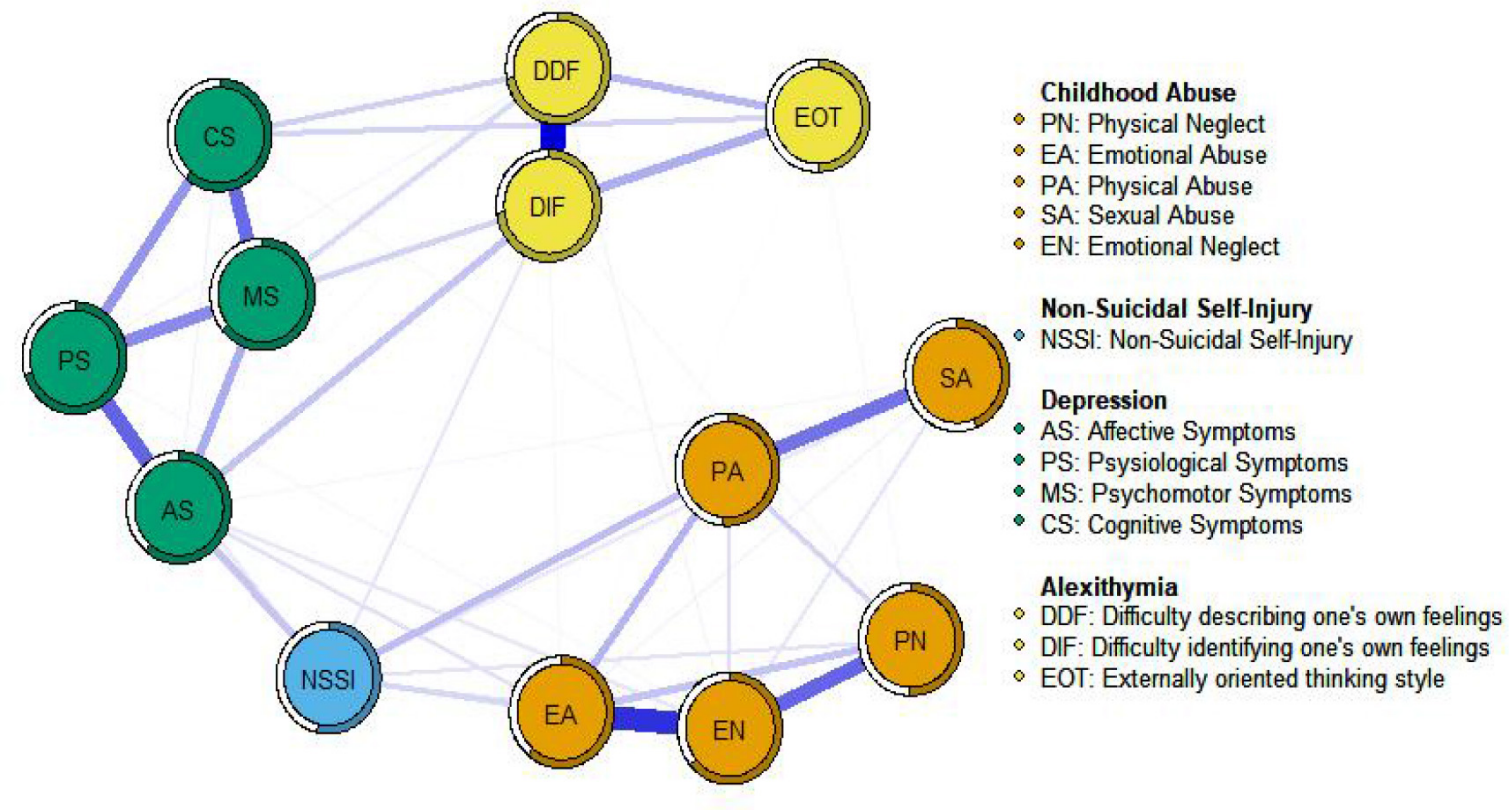
Network structure of childhood maltreatment-alexithymia-depression-NSSI symptoms. Purple edges represent positive correlations. The thickness of the edge reflects the magnitude of the correlation. Cut value = 0.05.

Furthermore, as an exploratory analysis, we constructed a symptom network to examine the micro-level interrelationships among different types of childhood maltreatment, specific dimensions of alexithymia, various depressive symptoms, and NSSI. Figure 2 illustrates the network structure of childhood maltreatment, alexithymia, depression, and NSSI. The strongest edges within the network were: DDF-DIF (weight = 0.57), EA-EN (weight = 0.47), PN-EN (weight = 0.35), and AS-PS (weight = 0.35). These strong associations may reflect tight internal integration among nodes within the same community, particularly within alexithymia and depression. Regarding bridge connections to NSSI, PA showed the strongest link from the childhood maltreatment community (weight = 0.15), AS from the depression community (weight = 0.13), and DIF from the alexithymia community (weight = 0.06). Thus, NSSI was primarily connected to the other symptom communities via PA (childhood maltreatment), AS (depression), and DIF (alexithymia). For inter-community bridges, the strongest connections were between EA and AS (childhood maltreatment-depression; weight = 0.07), EN and DDF (childhood maltreatment-alexithymia; weight = 0.03), and AS and DIF (depression-alexithymia; weight = 0.13). These cross-community pathways indicate that the depression and alexithymia communities are most strongly linked via the AS-DIF edge, while the childhood maltreatment community connects to depression via EA-AS and to alexithymia via EN-DDF. A full matrix of edge weights is provided in Supplementary Table S1. The stability of these estimates is supported by the relatively narrow bootstrapped 95% confidence intervals shown in Supplementary Figure S1, supporting the reliability of the edge weight estimates.

**Figure 3:**
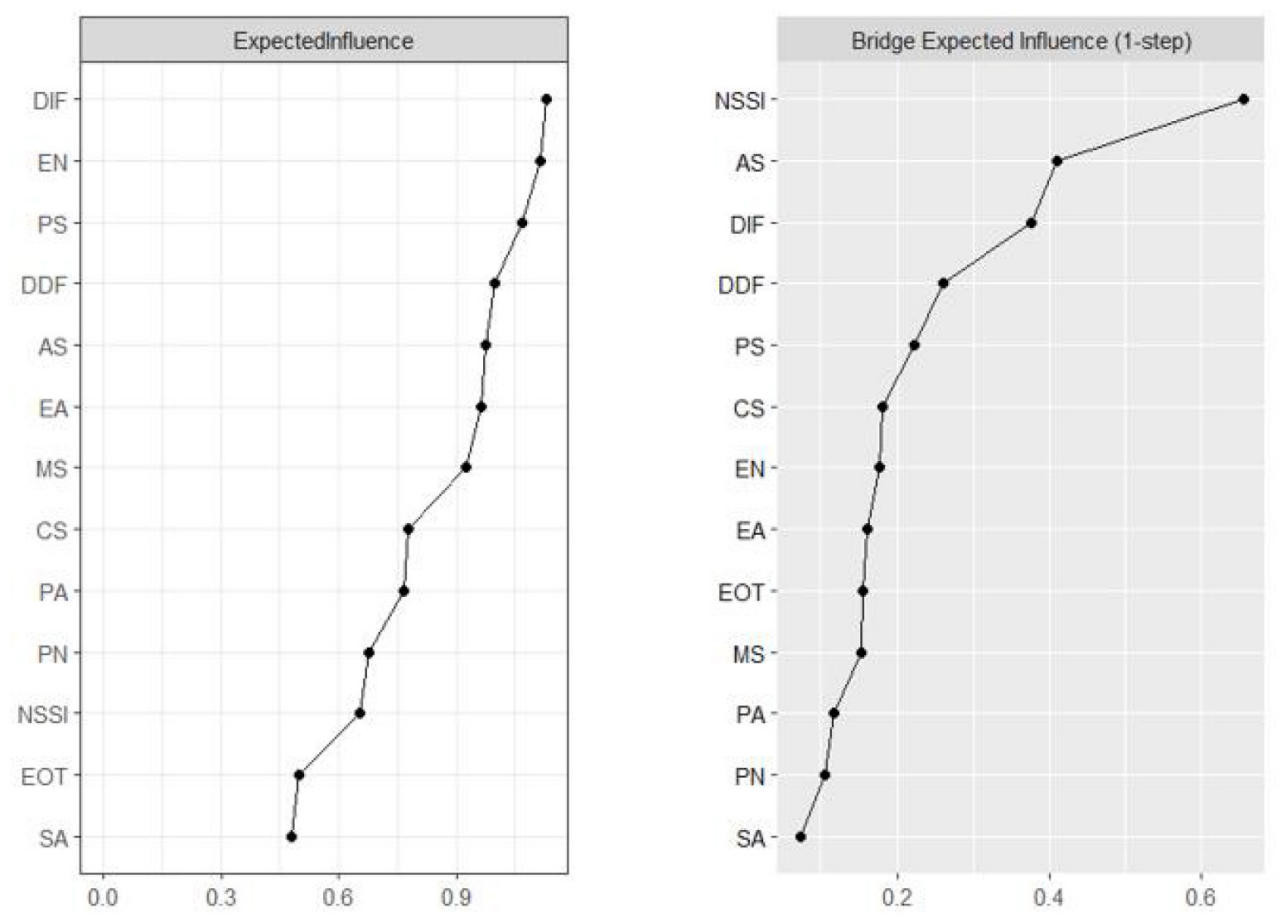
The node centrality (EI) and bridge centrality (BEI) of the maltreatment-alexithymia-depression-NSSI network (z-score).

The network's average predictability was 0.597, meaning that 59.7% of the variance in each node was explained by its neighboring nodes. This high level of shared variance contributes to a stable and trustworthy network structure. Predictability values for all nodes are listed in Supplementary Table S2. Figure 3 shows the node and bridge centrality, measured as z-scores, for the whole network. DIF displayed the highest node centrality (Expected Influence, EI = 1.129), indicating its dominant role within the network. This finding suggests that DIF may serve as a key connector linking childhood maltreatment, alexithymia, depression, and NSSI. NSSI had the highest bridge centrality (Bridge Expected Influence, BEI = 0.655), identifying it as the primary connector to other communities, followed by AS (BEI = 0.410) and DIF (BEI = 0.375). These findings highlight NSSI, AS, and DIF as the most critical bridge nodes, which may play a key role in connecting different symptom clusters. The strongest bridge connections were between NSSI and PA, and between AS and EA. This suggests that interventions targeting these two pathways may help alleviate the mutual reinforcement among symptoms, thereby reducing overall symptom severity. The complete EI and BEI values for all nodes are provided in Supplementary Table S3. The correlation stability coefficients for both node and bridge expected influence were 0.750, indicating high stability of the centrality indices (Supplementary Figures S2, S3). Bootstrapped difference tests for edge weights and node centrality are shown in Supplementary Figures S4, S5.

## 4 Discussion

Previous research on NSSI has tended to rely on either SEM or network analysis in isolation. The present study employed both approaches as complementary analytical tools: SEM for macro-level hypothesis testing and network analysis for exploratory micro-level examination. This dual-method approach was used to investigate the associations among childhood maltreatment, alexithymia, depression, and NSSI. SEM revealed positive associations between childhood maltreatment and NSSI, with alexithymia and depression serving as independent and sequential mediators. Specifically, individuals who reported greater childhood maltreatment also reported higher levels of alexithymia, depression, and NSSI. This macro-level analysis clarified the statistical pathways linking distal and proximal risk factors, thereby providing support for the proposed hypotheses. Given that all hypotheses were supported by SEM, we conducted network analysis as an exploratory complement to further elucidate the micro-level interrelationships among the study variables. Within this network, DIF emerged as the most central node, bridging childhood maltreatment, alexithymia, depression, and NSSI. Additionally, NSSI, AS, and DIF emerged as key bridge symptoms, connecting distinct symptom clusters and offering potential targets for intervention. Thus, network analysis complements SEM by identifying which specific symptoms function as critical hubs and bridges within the complex system of associations.

Our research found that childhood maltreatment may positively associated with NSSI among college students, aligning with existing literature indicating that a history of maltreatment is associated with an elevated risk of NSSI (Liu et al., 2021; Wang, 2024). While SEM analysis identified this macro-level association, the network analysis provided a more nuanced understanding by identifying which specific dimensions of childhood maltreatment were most strongly connected to NSSI. Specifically, PA showed the strongest direct connection to NSSI within the childhood maltreatment cluster, followed by EA. This finding suggests that, rather than operating as a homogeneous risk factor, the association between childhood maltreatment and NSSI in college students may be primarily driven by experiences of PA. This finding contrasts with Li Y. et al. (2024), who found EA to be the primary factor associated with NSSI among adolescents with major depressive disorder. The integration of SEM and network analysis helps contextualize this discrepancy by highlighting how the relative salience of different maltreatment dimensions may shift across development. In adolescence, a critical period for identity and emotional development, EA may directly damage self-esteem and emotional regulation (Pisani et al., 2013; Pilkington et al., 2024), making it a potent risk factor for NSSI. In contrast, for college students who have often left their family environment, the memory of PA as a concrete and objective traumatic event (Epstein and Bottoms, 2002; Barnett et al., 2024) may become more salient in the context of their new independence, strengthening its association with NSSI.

This study also found that alexithymia may mediate the relationship between childhood maltreatment and NSSI. This finding suggests that experiences of childhood maltreatment may disrupt normal emotional development, thereby impairing individuals' ability to effectively process and express emotions. When internal emotional distress cannot be adequately regulated or alleviated through adaptive means over time, individuals may resort to maladaptive and extreme coping responses. Consequently, they may engage in NSSI to manage stress, a pattern consistent with prior research (Wang, 2024; Li W. et al., 2024). This mediating role can be understood through several theoretical frameworks. According to the attention-appraisal model of alexithymia (Preece et al., 2017; Preece and Gross, 2023), individuals with high levels of alexithymia exhibit deficits in both the attention and evaluative stages of emotion processing. Childhood maltreatment, particularly emotional abuse and neglect, may disrupt the developmental capacity to learn how to recognize, understand, and express emotions effectively, thereby contributing to alexithymia (Brown et al., 2021). When faced with intense negative emotions, the inability to articulate this inner distress may lead to a buildup of emotional tension. In this state, NSSI may function as a maladaptive, non-verbal coping strategy. By creating physical pain, it may divert attention from or express otherwise ineffable emotional distress, thereby providing temporary emotional relief (Zetterqvist et al., 2013; Zetterqvist, 2015). Alexithymia is strongly associated with NSSI, primarily due to the characteristic emotional regulation deficits it entails. A core feature of alexithymia is impaired emotion regulation (Lai et al., 2026; Preece et al., 2022; Stasiewicz et al., 2012), which manifests as a tendency to use inhibitory strategies rather than cognitive reappraisal when coping with emotions (Preece et al., 2023). Accordingly, individuals with higher levels of alexithymia may be more likely to engage in NSSI as a maladaptive strategy for coping with emotional distress (Preece et al., 2017; Gross, 2015). The macro-level mediation analysis was complemented and refined by the micro-level network analysis, which identified the specific symptom driving this pathway. Specifically, DIF emerged as the most central node within the network, bridging childhood maltreatment, alexithymia, depression, and NSSI. This finding aligns with Gross and Jazaieri (2014) emotion regulation theory, which posits that recognizing and labeling emotions is a prerequisite for emotional regulation. The centrality of DIF suggests that the mediating role of alexithymia observed in the SEM model may be primarily attributed to DIF, rather than to difficulties in describing feelings or externally oriented thinking. In other words, childhood maltreatment may specifically impair the ability to identify emotional states, which in turn may disrupt emotion regulation and increase reliance on NSSI as a coping mechanism. This interpretation is consistent with recent evidence indicating that DIF is the most robust dimension of alexithymia in its associations with anxiety, depression, and perceived stress (Preece et al., 2024).

Although alexithymia has traditionally been viewed as a relatively stable trait, emerging evidence suggests that it may be ameliorated through clinical interventions targeting emotion regulation skills (Norman et al., 2019). The present findings refine this understanding by pinpointing DIF as a specific potential therapeutic target, suggesting that interventions enhancing emotional identification skills may be particularly effective in reducing the risk of NSSI.

The findings indicate that depression may mediate the relationship between childhood maltreatment and NSSI. While previous research has often characterized depression and NSSI as related internalizing and externalizing problems (Zhang et al., 2022), with a high correlation between NSSI and lifetime depression (He et al., 2023), our results clarify this link. In this study, we are more inclined to view depression as a state that is closely related to NSSI and has negative emotional characteristics. This was supported by Nock et al. (2010) experiential avoidance theory, which posits that individuals may use NSSI to achieve rapid emotional relief when they lack effective strategies for regulating negative emotions. The macro-level mediation analysis was further elucidated by the micro-level network analysis, which revealed the specific symptom-level connections underlying this pathway. The network analysis identified the strongest correlations between NSSI and PA, as well as between AS and EA. Specifically, the strong EA-AS connection suggests that the mediating role of depression may be partially explained by the link between EA and AS. Individuals with a history of prolonged EA may develop stable, negative self-cognition. They may internalize feelings of worthlessness and a belief that they are unworthy of care, which may increase their susceptibility to the AS of depression (Hamilton et al., 2013).

Moreover, the findings also indicate that alexithymia and depression may form a chain-mediating pathway between childhood maltreatment and NSSI. College students with a history of childhood maltreatment are more likely to report difficulties in emotion processing (i.e., alexithymia) and experience more intense negative affective states (i.e., depression). Such emotional distress was associated with a greater likelihood of engaging in NSSI as a maladaptive coping strategy that may provide rapid, albeit temporary, relief from intense negative emotions (Broadway-Horner et al., 2022). This is consistent with Li et al. (2023), who found that alexithymia mediated the link between childhood maltreatment and suicidal ideation in adolescents with major depression.

Although alexithymia and depression are often viewed as comorbid conditions, the present model conceptualizes them as distinct in emotional processing. This view is consistent with cognitive appraisal theory (Haaga et al., 1991), which posits that maladaptive cognitive schemas may contribute to increased negative affect and behavioral problems. Specifically, individuals with severe childhood maltreatment may tend to exhibit greater emotional inhibition in coping with interpersonal distress. Such patterns are associated with higher levels of alexithymia, a condition characterized by difficulties identifying and describing emotions (Ditzer et al., 2023; Gruhn and Compas, 2020). Alexithymia is associated with impairments in identifying one's own emotions and greater vulnerability to helplessness under stress, which are related to more severe depressive symptoms and an increased likelihood of NSSI (Zhou and Zhen, 2022; Valencia-Agudo et al., 2018). The macro-level chain mediation model was complemented and refined by the micro-level network analysis, which identified the specific symptoms driving each link in this pathway. Network analysis revealed that DIF within the alexithymia community and AS within the depression community emerged as key bridge nodes connecting to NSSI. Additionally, DIF served as the most central node in the overall network, bridging childhood maltreatment, alexithymia, depression, and NSSI. These findings suggest that future efforts to identify and intervene in NSSI among college students may consider giving priority to addressing DIF and AS.

## 5 Limitation

This study integrated SEM with network analysis to explore the associations linking childhood maltreatment, alexithymia, depression, and NSSI. However, several limitations should be acknowledged and addressed in future research. First, despite identifying a chain-mediating effect of alexithymia and depression through SEM, the cross-sectional design of our data limits causal interpretation. Future research requires longitudinal designs with repeated measurements to capture the temporal dynamics and potential reciprocity among these constructs. Second, although the indirect effects were statistically significant, the effect sizes were relatively small. This finding suggests that the associations between childhood maltreatment, alexithymia, depression, and NSSI may be influenced by additional factors not accounted for in the current model. Future studies should explore these potential influences. Third, the reliance on self-report measures may introduce biases such as imperfect recall and social desirability, particularly for sensitive topics, potentially compromising data accuracy. Future studies should consider incorporating objective measures to triangulate these findings. Fourth, the network structure estimated in this study is based on a specific sample, and its stability and generalizability require replication and validation across different cultural contexts, clinical populations, and larger samples. Moreover, the cross-sectional nature of the network analysis precludes causal inferences, as centrality indices reflect correlational structures and are not directly indicative of clinical intervention targets. Future research should employ longitudinal designs and causal analytic approaches to determine whether DIF exerts a causal influence on symptom dynamics and represents a precise intervention target. Finally, the overrepresentation of female participants in this study may limit the generalizability of the findings to males. Future research should employ more balanced samples to further examine and extend the applicability of these results across genders.

## 6 Practical implications

This study reveals that childhood maltreatment is significantly associated with NSSI among college students, in which alexithymia and depression may serve as chain mediators. This finding suggests that maltreatment may persist in its effects and is associated with long-term psychological adjustment and adverse outcomes including NSSI. Consequently, early identification and intervention are paramount. A collaborative, multi-system approach is recommended, wherein schools, communities, and families establish a monitoring network to enhance vigilance against childhood maltreatment. Schools should implement regular mental health screenings to track students' emotional and behavioral wellbeing. Upon detection of abuse signs, structured intervention protocols must be activated promptly to provide comprehensive support, including psychological counseling and legal assistance for affected individuals. Such proactive measures are critical for mitigating psychological trauma, reducing the risk of alexithymia and depression, and ultimately preventing the development of NSSI.

Network analysis reveals that DIF is the most central node within the network, with DIF, NSSI, and AS serving as pivotal bridges connecting clusters within the network. This underscores the critical role of emotional recognition and expression in preventing NSSI. Therefore, integrating emotional education into the school curriculum represents an effective pathway to enhance pupils' emotional competence. Curriculum design could include dedicated modules that teach students to identify diverse emotions, understand their triggers, and master effective regulation strategies. Expanded affective education activities would provide opportunities to practice navigating intense feelings. Concurrently, teachers should embed emotional learning into daily instruction by observing students' emotional responses and providing timely support. Furthermore, as a primary environment for emotional development, families should cultivate a warm and supportive atmosphere. Parents are encouraged to maintain open communication, validate emotional expression, and guide their children in managing emotional challenges. Through school-family collaboration, students' emotional awareness and expression skills can be improved, reducing depressive symptoms, thereby the incidence of NSSI can be effectively reduced.

Moreover, college students are at a critical developmental stage, navigating significant pressures from academics, social life, and future employment, which renders them vulnerable to psychological issues. The findings of this study highlight the need for an integrated support system to assist students with a history of childhood maltreatment, alexithymia, and depressive symptoms.

## 7 Conclusions

This study employs SEM to identify the associations among childhood maltreatment, alexithymia, depression, and NSSI. Additionally, network analysis was conducted as an exploratory approach to investigate the micro-level interconnections among symptoms of these constructs. The findings show that alexithymia and depression may mediate the association between childhood maltreatment and NSSI. Specifically, they may function as chain mediators in this relationship. Network analysis reveals that DIF is a central symptom, while NSSI, AS and DIF served as key bridges connecting the constructs. We highlight the critical role of emotional processing deficits in NSSI and indicate that identification and intervention efforts for NSSI among college students may consider giving priority to addressing these core emotional issues.

## Data Availability

The raw data supporting the conclusions of this article will be made available by the authors, without undue reservation.
