# Supplementary material for: Childhood maltreatment and non-suicidal self-injury in college students: chain mediation by alexithymia and depression along with a symptom-level network analysis
Source: Front Psychol. 2026 Jul 8;17:1751101. doi: 10.3389/fpsyg.2026.1751101 (PMC13388171; doi:10.3389/fpsyg.2026.1751101)
Supplement: Supplementary file 1 [file Supplementary_file_1.docx]

**Supplemental Material**

1. Table S1. All edge weights within the CA-Alex-Dep.-NSSI network
2. Table S2. All node predictability of the CA-Alex-Dep.-NSSI network
3. Table S3. All node EI and BEI of the CA-Alex-Dep.-NSSI network
4. Figure S1. Accuracy of edge weights
5. Figure S2. Stability of node expected influences
6. Figure S3. Stability of bridge expected influences
7. Figure S4. Bootstrapped difference test for node bridge expected influences
8. Figure S5. Bootstrapped difference test for edge weights

**Table S1. All edge weights within the CA-Alex-Dep.-NSSI network**

|  | **PN** | **EA** | **PA** | **SA** | **EN** | **NSSI** | **AS** | **PS** | **MS** | **CS** | **DDF** | **DIF** | **EOT** |
| --- | --- | --- | --- | --- | --- | --- | --- | --- | --- | --- | --- | --- | --- |
| **PN** | 0 |  |  |  |  |  |  |  |  |  |  |  |  |
| **EA** | 0.133 | 0 |  |  |  |  |  |  |  |  |  |  |  |
| **PA** | 0.094 | 0.167 | 0 |  |  |  |  |  |  |  |  |  |  |
| **SA** | 0.000 | 0.039 | 0.313 | 0 |  |  |  |  |  |  |  |  |  |
| **EN** | 0.346 | 0.465 | 0.074 | 0.053 | 0 |  |  |  |  |  |  |  |  |
| **NSSI** | 0.062 | 0.091 | 0.146 | 0.048 | 0.074 | 0 |  |  |  |  |  |  |  |
| **AS** | 0.000 | 0.066 | 0.000 | 0.021 | 0.053 | 0.127 | 0 |  |  |  |  |  |  |
| **PS** | 0.003 | 0.000 | -0.008 | 0.000 | 0.020 | 0.050 | 0.349 | 0 |  |  |  |  |  |
| **MS** | 0.000 | 0.000 | 0.000 | 0.000 | 0.000 | 0.000 | 0.184 | 0.260 | 0 |  |  |  |  |
| **CS** | 0.000 | 0.000 | -0.021 | 0.000 | 0.000 | 0.000 | 0.031 | 0.236 | 0.330 | 0 |  |  |  |
| **DDF** | 0.000 | 0.000 | 0.000 | 0.000 | 0.030 | 0.000 | 0.013 | 0.030 | 0.084 | 0.105 | 0 |  |  |
| **DIF** | 0.022 | 0.017 | 0.000 | 0.004 | 0.000 | 0.057 | 0.130 | 0.098 | 0.047 | 0.000 | 0.573 | 0 |  |
| **EOT** | 0.018 | -0.012 | 0.000 | 0.000 | 0.000 | 0.000 | 0.000 | 0.029 | 0.023 | 0.097 | 0.163 | 0.181 | 0 |

**Table S2. All node predictability of the CA-Alex-Dep.-NSSI network**

|  | Variable | *R*^2^ |
| --- | --- | --- |
| **1** | PN | 0.506 |
| **2** | EA | 0.612 |
| **3** | PA | 0.514 |
| **4** | SA | 0.436 |
| **5** | EN | 0.636 |
| **6** | NSSI | 0.536 |
| **7** | AS | 0.611 |
| **8** | PS | 0.699 |
| **9** | MS | 0.642 |
| **10** | CS | 0.614 |
| **11** | DDF | 0.72 |
| **12** | DIF | 0.731 |
| **13** | EOT | 0.5 |

**Table S3. All node EI and BEI of the CA-Alex-Dep.-NSSI network**

|  | **EI** | **BEI** |
| --- | --- | --- |
| **PN** | 0.678 | 0.105 |
| **EA** | 0.965 | 0.161 |
| **PA** | 0.766 | 0.117 |
| **SA** | 0.479 | 0.073 |
| **EN** | 1.115 | 0.176 |
| **NSSI** | 0.655 | 0.655 |
| **AS** | 0.974 | 0.410 |
| **PS** | 1.066 | 0.222 |
| **MS** | 0.927 | 0.154 |
| **CS** | 0.778 | 0.182 |
| **DDF** | 0.997 | 0.261 |
| **DIF** | 1.129 | 0.375 |
| **EOT** | 0.499 | 0.154 |


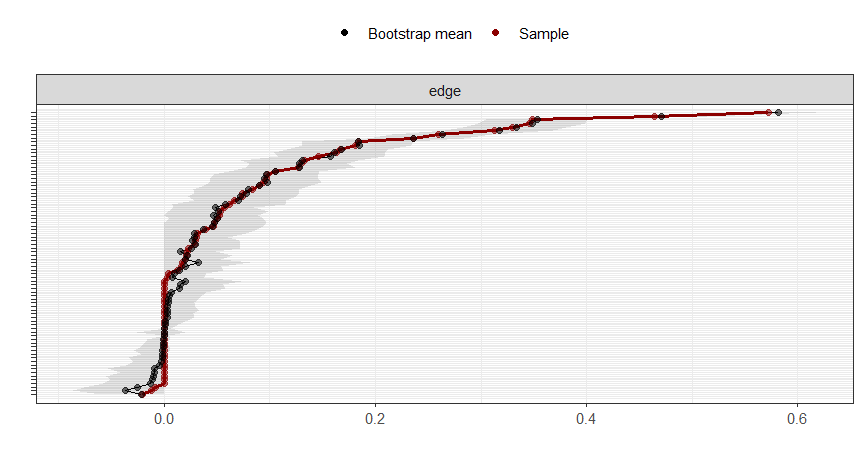


**Figure S1**. Accuracy of edge weights

Note: The red line depicts the sample edge weights and the gray bar depicts the bootstrapped confidence interval.


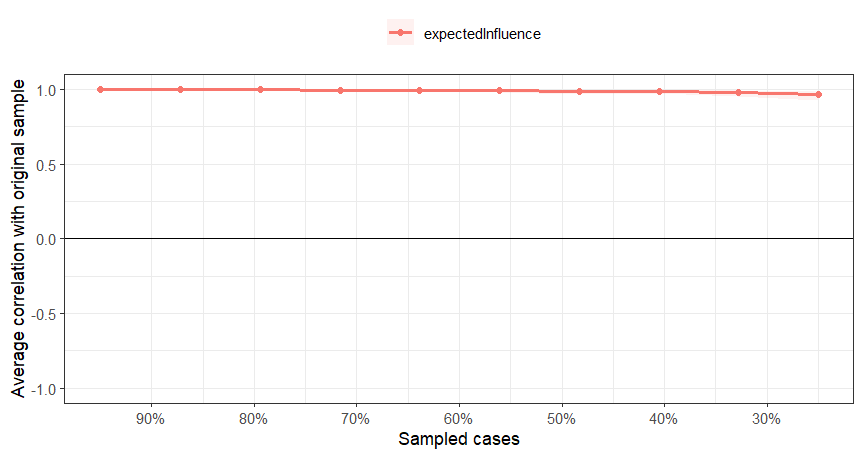


**Figure S2**. Stability of node expected influences


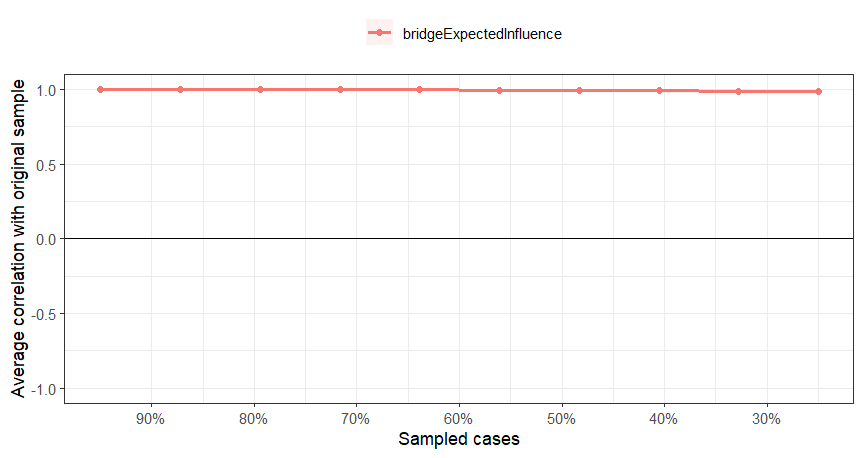


**Figure S3**. Stability of bridge expected influences


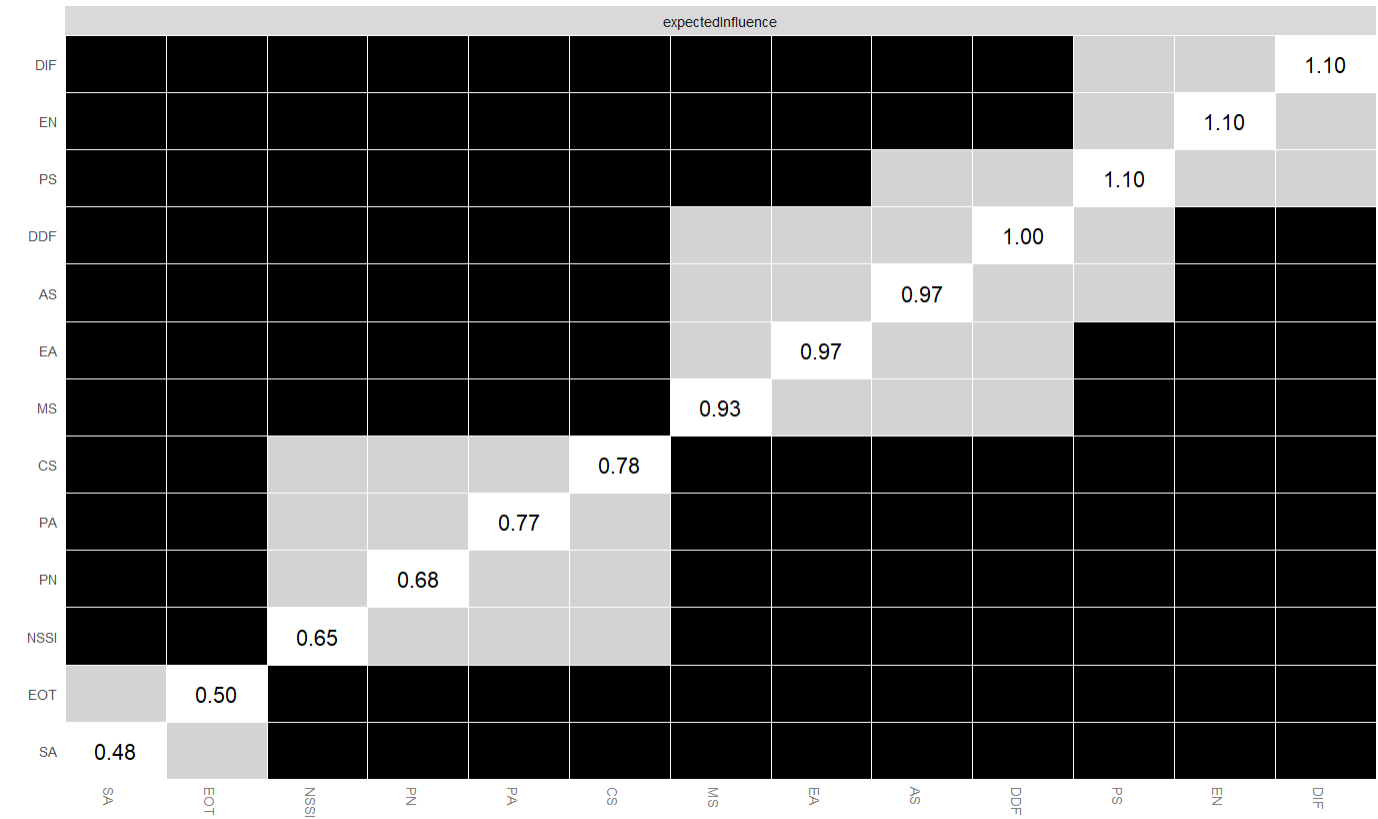
**Figure S4**. Bootstrapped difference test for node bridge expected influences


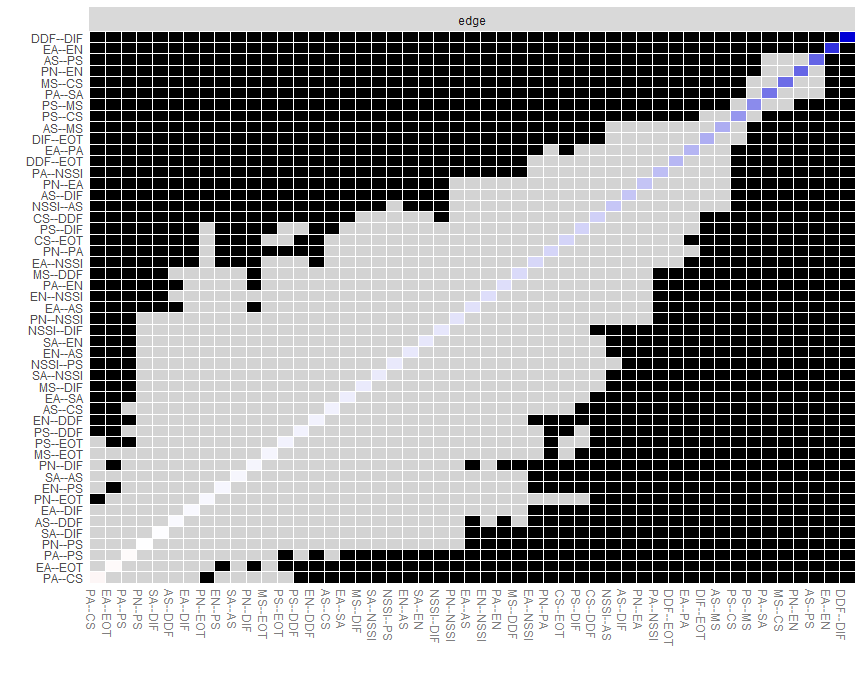


**Figure S5.** Bootstrapped difference test for edge weights
